# The Effects of *Ocimum basilicum *Extract and Its Constituent, Rosmarinic Acid on Total and Differential Blood WBC, Serum Levels of NO, MDA, Thiol, SOD, and CAT in Ovalbumin Sensitized Rats

**Published:** 2018

**Authors:** Naeima Eftekhar, Ali Moghimi, Mohammad Hossein Boskabady

**Affiliations:** a *Department of Biology, Faculty of Science, Ferdowsi University of Mashhad, Mashhad, Iran. *; b *Neurogenic Inflammation Research Center, Mashhad University of Medical Sciences, Mashhad, Iran.*; c *Department of Physiology, School of Medicine, Mashhad University of Medical Sciences, Mashhad, Iran.*

**Keywords:** Ocimum basilicum, Rosmarinic acid, White blood cells, Oxidant and antioxidant markers, Sensitized rats

## Abstract

The effects of *Ocimum basilicum (O. basilicum)* and its constituent, rosmarinic acid, on total and differential blood WBC, serum levels of NO_2_, NO_3_, MDA, thiol, SOD, and CAT in sensitized rats were examined. The study was performed in control animals (group C) and eight groups of sensitized rats to ovalbumin which were given drinking water alone (group S), drinking water containing three concentrations of *O. basilicum* (O; 0.75, 1.5 and 3.0 mg/mL), three concentrations of rosmarinic acid (R; 0.125, 0.25 and 0.5 mg/mL) and dexamethasone (1.25 μg/mL), (n = 6 for R treated and n = 8 for other groups). Total and differential WBC as well as serum concentrations of oxidant and antioxidant biomarkers were measured in all groups. Serum levels of oxidant biomarkers, total WBC count, percentages of eosinophils and neutrophils were significantly increased but other measured parameters except monocytes were significantly decreased in group S compared to group C. Serum levels of oxidant biomarkers, total WBC count, percentages of eosinophils, neutrophils and monocytes were significantly decreased but other measured parameters were significantly increased in treated S groups with dexamethasone, extract, and rosmarinic acid compared to group S. The effects of the extract and rosmarinic acid on some measured variables were significantly higher than the effect of dexamethasone treatment. These results showed the effect of *O. basilicum* and its constituent, rosmarinic acid on inflammatory and oxidant parameters in sensitized rats which was comparable or even more potent than dexamethasone at used concentrations.

## Introduction

Asthma is a chronic inflammatory airway disease. It is characterized by airway hyperresponsiveness (AHR), multicellular inflammation and airway obstruction which is accompanied by intermittent episodes of wheeze and cough (1)**. **The main pathological characteristic feature of asthma is respiratory tract inflammation (2). It has been shown that inflammation driven by increased oxidative stress occurs in the airways of patients with asthma (3). Lungs and blood have many enzymatic and non-enzymatic antioxidants like glutathione peroxidase (GPx), superoxide dismutase (SOD), catalase (CAT), heme oxygenase, thioredoxin, peroxiredoxin, glutathione, carotenoids, vitamins E, A and C which all play a role in oxidant toxicity. An oxidant-antioxidant imbalance and loss of antioxidant biomarkers is found in asthma (4). Increased total WBC and eosinophil count in sensitized animals (5) and asthmatic patients (6) were also shown. 

Plants have been used for medical treatments through much of human history. This tradition seems to be the basis of modern medicine, as many of the pharmaceutical drugs are derived from plants origin (7). *Ocimum basilicum *(*O. basilicum*) or Basil is an annual herb of the Lamiaceae family. It is consumed as a seasoning in dry and fresh form. It is native to tropical Asia, but it is now cultivated all over the world. *O. basilicum* derived compounds include mainly triterpenoids, polyphenols, steroids, phenylpropanoids, and rosmarinic acid which is one of its main phenolic compounds (8). 

The therapeutic and medicinal values of this plant are the subjects of many researches ant its safe­ty in animal and human models has been confirmed (9). Anti-HIV (10) anti-aging and anti-cancer (11) effects of *O. basilicum* have been reported. The relaxant effect of this plant on tracheal smooth muscle was observed in our previous study (12). Fathiazad and colleagues showed that cardioprotective effect of *O. basilicum* is correlated with its antioxidant activity (13). Antioxidants are compounds which restrain oxidative damage through variable mechanisms such as reacting with free radicals, chelating catalytic metals, and acting as oxygen scavengers. There is an increasing interest to elucidate the association of different antioxidants with stress-related conditions such as inflammatory disease (14). Anti-inflammatory and antioxidant properties of Osmium genus have been documented in some studies (15-16). Rosmarinic acid is one of the main phenolic compounds in sweet basil which has pharmacological properties such as antioxidant (17) antiviral, antimicrobial, and anti-inflammatory (18) effects. 

With regard to respiratory tract inflammation in asthma and due to anti-inflammatory and antioxidant properties of *O. basilicum* and rosmarinic acid, the effects of hydroalcoholic extract of the plant and its constituent, rosmarinic acid on total and differential blood WBC count as well as serum levels of NO_2_, NO_3_, MDA, thiol, SOD and CAT in sensitized rats were examined in the present study.

## Experimental

*Plant and extract*

*Ocimum basilicum *was collected from the plant garden of School of Pharmacy, Mashhad city, Khorasan Razavi province, Iran, and identified by Mr. Joharchi and a specimen was preserved in the herbarium of school of agriculture, Ferdowsi University of Mashhad with herbarium number 12937061. The leaves of this plant were dried in shadow and grinded. The extract was obtained by maceration method. For preparation of hydroethanolic extract, 100 g of *O. basilicum *powder in 1000 mL ethanol 70% was macerated in laboratory temperature for 72 h. To prepare the dry extract, the solvent was evaporated by a rotary evaporator. The yield extract was 19%. Finally, the extract concentration was adjusted to 10 mg/mL by adding distilled water to the dried extract.

*Animal sensitization and animal groups*

Male Wistar rats weighing 220 ± 250 g were sensitized on days 1, 2 and 3 by 1 mg/kg intraperitoneal injections of ovalbumin (OVA) in 0.9% sterile saline containing 100 mg Al (OH)_3_ as adjuvant. On days 6, 9, 12, 15, 18, and 21, animals were exposed to 1% ovalbumin aerosol produced by a DeVilbiss PulmoSonic nebulizer (DeVilbiss Health Care Ltd., Feltham, U.K.) for 20 min with air flow of 8 lit/min. Challenges took place in a 0.8 m^3^ chamber, with animal normal-breathing. The rats were housed in a caging system receiving clean filtered air (Maximiser, Thorens Caging System Inc., Hazleton, PA, U.S.A.) with water and food available *ad libitum* during experimental period (19). In addition, the temperature was maintained at 22 ± 2 ºC on a 12 h light/dark cycle.

Nine groups of animals were studied in random order as fallows (n = 6 for R treated groups and n = 8 for other groups):

(1) Non sensitized control animals (saline was used instead of OVA for IP injection and inhalation) (group C)

(2) Untreated sensitized animals, (group S)

(3) Sensitized group treated with dexamethasone 1.25 μg/ mL, (group D)

(4-6) Sensitized groups treated with *O. basilicum *extract at 3 concentrations of 0.75, 1.5 and 3.0 mg/mL in animal’s drinking water during sensitization period, (Groups O 0.75, O 1.5 and O 3.0) (20).

(7-9) Sensitized groups treated with rosmarinic acid at 3 concentrations of 0.125, 0.25, and 0.50 mg/mL in animal’s drinking water during sensitization period, (Groups R 0.125, R 0.25 and R 0.50) (21).

Each rat drank averagely 40 mL/day drinking water and there wasn’t significant difference in the used drinking water between different groups.

*Preparation of blood sample*

Animals were sacrificed at day 22 of experiment by ketamine. Five mL blood sample was taken by cardiac puncture immediately after sacrificing and exposing the animal›s chest. For total and differential white blood cells measurements, two ml of blood sample was collected into the test tube containing anticoagulant EDTA. For measurement of oxidant, antioxidant markers, blood samples were collected in to test tube and placed at room temperature for 1 h. The samples were then centrifuged at 3500 g for 10 min. The supernatant was collected and immediately stored at −70 °C until analyzed.

*Estimation of serum oxidant levels*

*Nitric Oxide (NO)*

The total stable oxidation products of NO me­tabolism (NO_2_-/NO_3_-) of serum supernatant were as­sessed using a Griess reagent. The frozen serum was allowed to thaw and to reach a temperature of 25 °C that was fol­lowed by being deproteinized by zinc sulfate solu­tion (Sigma, America). The liquefied serum was then centrifuged at 12000 g for 10 min. Ali­quots (300 μL) of the clear supernatant was mixed with Griess reagents including 300 μL SULF (2% w/v, Sigma, America) in 5% HCl and 300 μL NEDD (0.1% w/v, Sigma, America) in H_2_O in a test tube, while for the reduction of nitrate to ni­trite, 300 μL saturated solutions of vanadium (III) chloride (VCl_3_; Sigma, America) in 1 M HCl was added and incubated for 2 h at 30 °C in the dark. Then, the absorbance of samples was meas­ured at 540 nm against a blank containing the same concentrations of ingredients but no biological sample. Linear regression was used to determine NO concentration from standard curve of NaNO_2_. The final results were expressed as μmol (22).

*Malondialdehyde (MDA)*

Malondialdehyde (MDA) reacts with thiobarbituric acid (TBA) as a thiobarbituric acid reactive substance (TBARS) to produce a red colored complex which has peak absorbance at 535 nm. Two mL from reagent of TBA/trichloroacetic acid (TCA)/HCl was added to 1 mL of serum supernatant and the solution was heated in a water bath for 40 min. After cooling, the whole solutions were centrifuged within 1,000×g for 10 min. The absorbance was measured at 535 nm (23). 

*Estimation of serum antioxidant levels*

*Total thiol*

Total thiol concentration was measured using DTNB reagent which reacts with the thiols to produce a yellow colored complex which has a peak absorbance at 412 nm. Briefly, 1 mL Trisethylenediaminetetraacetic acid (EDTA) buffer (pH 8.6) was added to 50 μL serum supernatant in 1 mL cuvettes and sample absorbance was read at 412 nm against Tris-EDTA buffer alone (A1). Then 20 μL DTNB reagents (10 mmol/L in methanol) were added to the mixture and after 15 min (stored in laboratory temperature) the sample absorbance was read again (A2). The absorbance of DTNB reagent was also read as a blank (B). Total thiol concentration (mmol/L) was calculated from the following equation (24).

Total thiol concentration (mmol/L) = (A2–A1–B)×1.07/0.05×13.6.

*Superoxide dismutase (SOD) activity*

A colorimetric assay involving generation of superoxide by pyrogallol auto-oxidation and the inhibition of superoxide-dependent reduction of the tetrazolium dye, MTT (3-(4,5-dimethylthiazol-2-yl) 2, 5-diphenyltetrazolium bromide) to its formazan by SOD was measured at 570 nm (25). One unit of SOD activity was defined as the amount of enzyme causing 50% inhibition in the MTT reduction rate.

*Catalase (CAT) activity*

CAT activity was estimated by the rate constant, k, (dimension: s-1, k) of hydrogen peroxide decomposition (26). The decrease in absorbance at 240 nm per minute and the rate constant of the enzyme was determined. Activities were expressed ask (rate of constant) per liter.

*White blood cells count*

Two mL blood sample was collected into the test tube containing anticoagulant EDTA immediately after sampling. Total white blood cells (WBC) were counted in duplicate in a hemocytometer (in a Burker chamber) after staining of the blood with Turk solution (1:10 dilution). The Turk solution contained 1 mL of glacial acetic acid, 1 mL of gentian violet solution 1% and 100 mL distilled water. For differential WBC counting a thin slide of the blood was prepared and stained with Wright-Giemsa›s. Differential cell analysis was carried out under a light microscope according to staining and morphological criteria, by counting 100 cells and the percentage of each cell type was calculated.

*Statistical analysis*

The results were presented as means ± SEM. The data of treated groups were compared to untreated group and also control group using one way analysis of variance (ANOVA) with Tukey-Kramer’s post-test. Comparisons between the data of three concentrations of extract and rosmarinic acid were also performed using (ANOVA) with Tukey-Kramer’s post-test. Significance was considered at *p < *0.05. The statistical analyses were performed using InStat (GraphPad Software, Inc, La Jolla, USA).

## Results

Serum NO_2_, NO_3_, MDA, thiol, SOD, CAT concentrations, total and differential WBC counts in untreated ant treated sensitized rats

**Figure 1:**
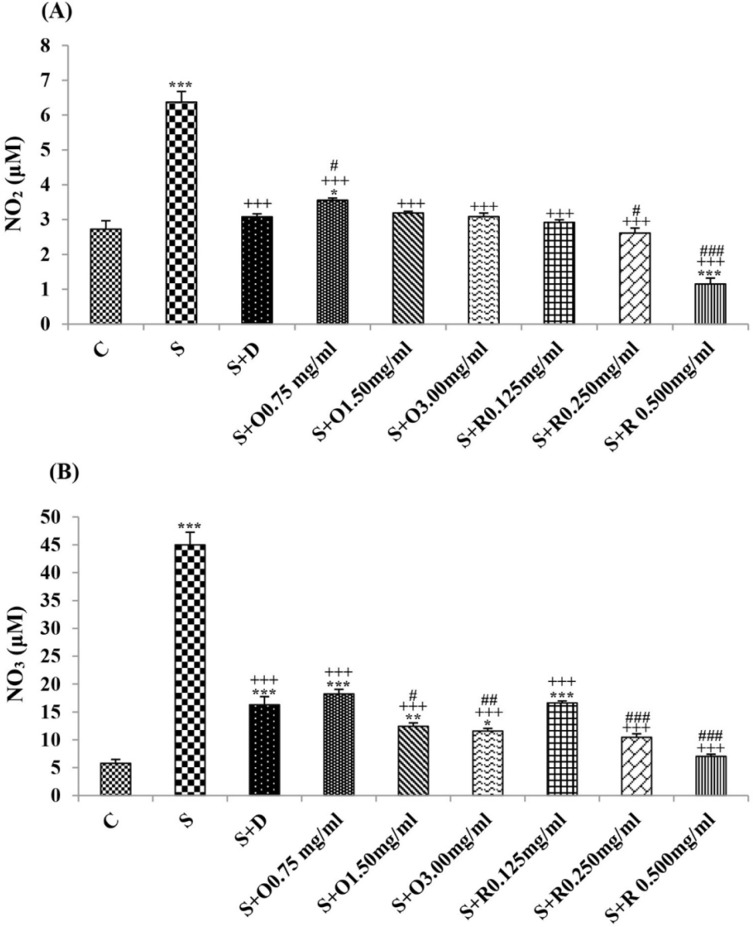
The levels of serum (A) NO_2_ (μM) and (B) NO_3_ (μM) in control rats (C), sensitized animals (S), S treated with dexamethasone (S + D), three concentrations of *O. basilicum* (S + O) and three concentrations of rosmarinic acid (S + R), (n = 6 for R treated groups and n = 8 for other groups).

**Figure 2:**
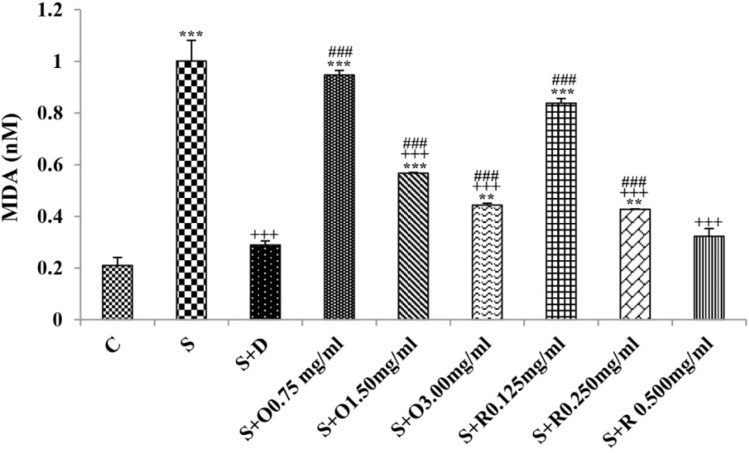
The levels of serum malondialdehyde (MDA) (nM) in control rats (C), sensitized animals (S), S treated with dexamethasone (S + D), three concentrations of *O. basilicum* (S + O) and three concentrations of rosmarinic acid (S + R), (n = 6 for R treated groups and n = 8 for other groups).

**Figure 3:**
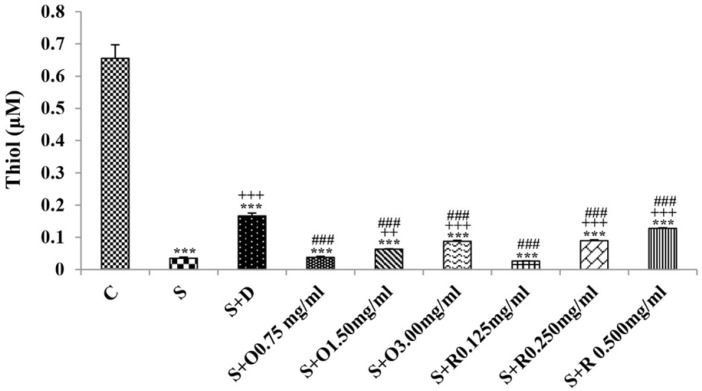
The levels of serum thiol (μM) in control rats (C), sensitized animals (S) S treated with dexamethasone (S + D), three concentrations of *O. basilicum* (S + O) and three concentrations of rosmarinic acid (S + R), (n = 6 for R treated groups and n = 8 for other groups).

**Figure 4:**
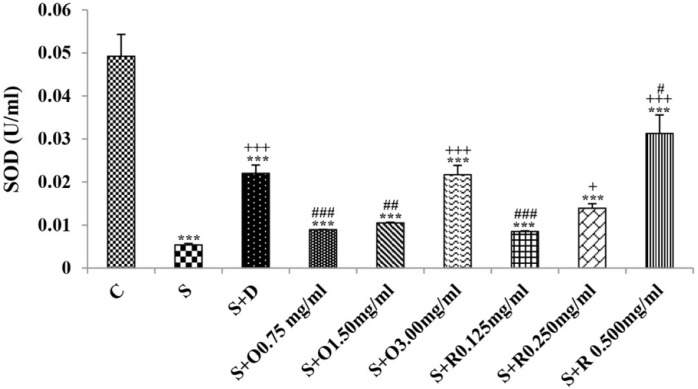
The levels of serum superoxide dismutase (SOD) (U/mL) in control rats (C), sensitized animals (S), S treated with dexamethasone (S + D), three concentrations of *O. basilicum* (S + O) and three concentrations of rosmarinic acid (S + R), (n = 6 for R treated groups and n = 8 for other groups)

**Figure 5:**
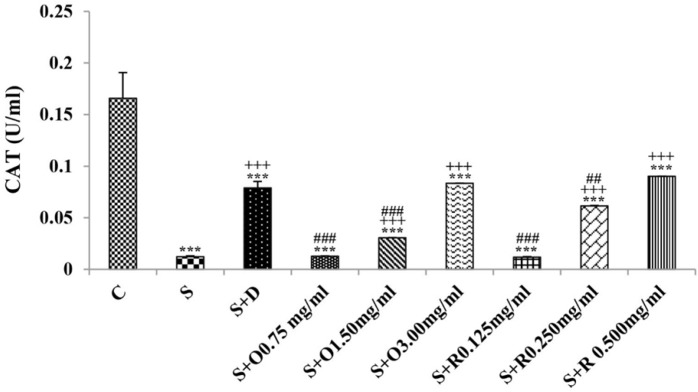
The levels of serum catalase (CAT) (U/mL) in control rats (C), sensitized animals (S), S treated with dexamethasone (S + D) three concentrations of *O. basilicum* (S + O) and three concentrations of rosmarinic acid (S + R), (n = 6 for R treated groups and n = 8 for other groups)

**Figure 6:**
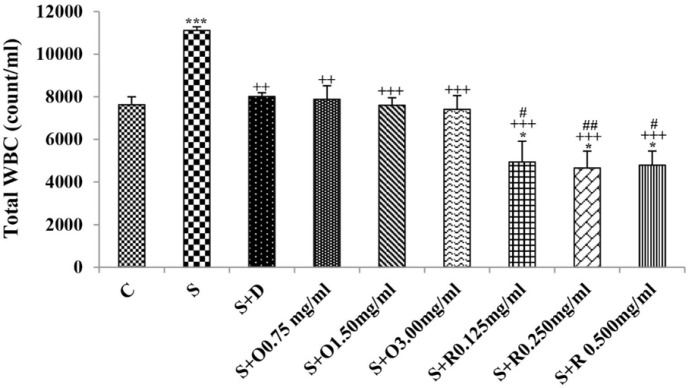
Total WBC number in one mL blood of control rats (C), sensitized animals (S), S treated with dexamethasone (S + D), three concentrations of *O. basilicum* (S + O) and three concentrations of rosmarinic acid (S + R), (n = 6 for R treated groups and n = 8 for other groups).

**Figure 7:**
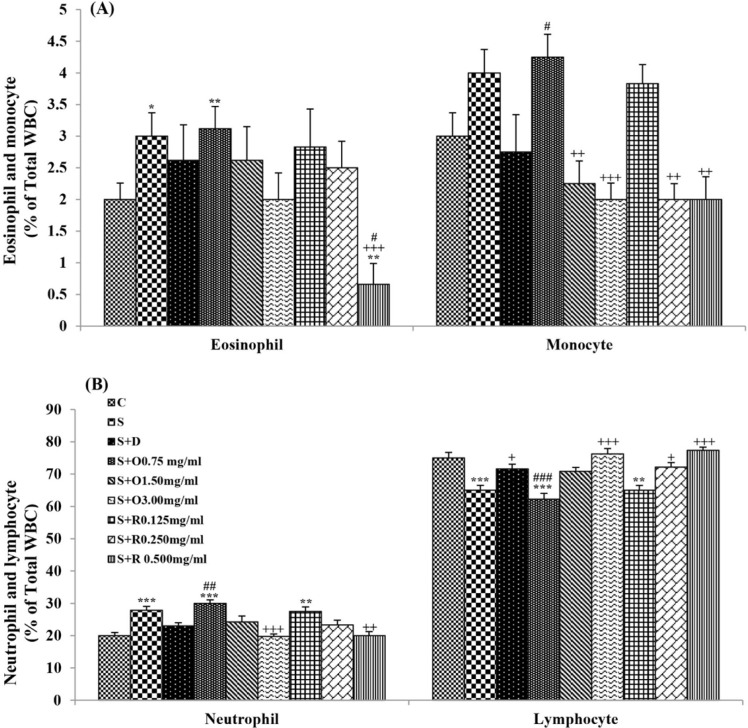
Percentages (mean ± SEM) of (A) eosinophil and monocyte, and (B) neutrophil and lymphocyte, in the blood of control rats (C), sensitized animals (S), S treated with dexamethasone (S + D), three concentrations of *O. basilicum* (S + O) and three concentrations of rosmarinic acid (S + R), (n = 6 for R treated groups and n = 8 for other groups).

Serum levels of NO_2_, NO_3_ and MDA, total WBC count, percentages of eosinophils, and neutrophils were significantly increased but serum levels of CAT, SOD, thiol, and percentage of lymphocytes were significantly decreased in sensitized compared to control group (*p *< 0.05 for eosinophils and *p *< 0.001 for other cases) (Figures 1-7).

Statistical significance for the differences between the data of S, S + D, S + O and S + R versus C: *: *p *< 0.05, **: *p *< 0.01, ***: *p *< 0.001.

Statistical significance for the differences between the data of S + D, S + O and S + R versus S: +++: *p *< 0.001.

Statistical significance for the differences between the data of S + O and S + R versus S + D: #: *p *<0.05, ##: *p *< 0.01, ###: *p *< 0.001. The statistical comparisons were made using (ANOVA) with Tukey-Kramer’s post-test.

Statistical significance for the differences between the data of S, S + D, S + O and S + R versus C: **: *p *< 0.01, ***: *p* < 0.001.

*Statistical significance for the differences between the data of S + D, S + O and S + R versus S: +++: *p *< 0.001. Statistical significances for the difference between the data of S + O and S + R versus S + D: ###:* p *< 0.001. The statistical comparisons were made using (ANOVA) with Tukey-Kramer’s post-test.*

Treatment of sensitized animals with all concentrations of the extract lead to significant decrease in serum concentrations of NO_2_, NO_3_ and total WBC count and also treatment with its two higher concentrations (1.5 and 3.0 mg/mL) caused reduction in MDA value and monocytes percentage but increased thiol and CAT values. Moreover, treatment with its highest concentration (3.0 mg/mL) resulted in reduction of neutrophils percentage but enhanced SOD value and lymphocytes percentage compared to untreated sensitized group (*p *< 0.01 to *p *< 0.001), (Figures 1-7). However, there were significant differences between control groups and sensitized animals treated with all concentrations of the extract on NO_3_, MDA, thiol, SOD, and CAT values (*p *< 0.05 to *p *< 0.001), (Figures 1-5). In addition, NO_2_ value (*p *< 0.05) as well as eosinophils, neutrophils and lymphocytes percentages (*p *< 0.01 to *p *< 0.001) were also significantly different between treated animals with low concentration of the extract (0.75 mg/mL) and control group (Figures 1 and 7).

Statistical significance for the differences between the data of S, S + D, S + O and S + R versus C: ***: p *< 0.001. Statistical significance for the differences between the data of S + D, S + O and S + R versus S: ++: *p *< 0.01, +++: *p *< 0.001. Statistical significance for the differences between the data of S + O and S + R versus S + D: ###: *p *< 0.001. The statistical comparisons were made using (ANOVA) with Tukey-Kramer’s post-test.*

Statistical significance for the differences between the data of S, S + D, S + O and S + R versus C: ***: *p*<0.001. Statistical significance for the differences between the data of S + D, S + O and S + R versus S: +: *p *< 0.05, +++: *p *< 0.001. Statistical significance for the differences between the data of S + O and S + R versus S + D: #: *p *< 0.05, ##: *p *< 0.01, ###: *p *< 0.001. The statistical comparisons were made using (ANOVA) with Tukey-Kramer’s post-test.

Statistical significance for the differences between the data of S, S + D, S + O and S + R versus C: ***: *p *< 0.001.

Statistical significance for the differences between the data of S + D, S + O and S + R versus S: +++: *p *< 0.001.

*Statistical significance for the differences between the data of S + O and S + R versus S + D: ##: *p *<0.01, ###: *p*<0.001. The statistical comparisons were made using (ANOVA) with Tukey-Kramer’s post-test.*

Treatment of sensitized animals with all concentrations of rosmarinic acid also resulted in significant decrease in serum concentrations of NO_2_, NO_3,_ and total WBC, treatment with its two higher concentrations (0.25 and 0.5 mg/mL) lead to reduction of MDA value and monocytes percentage but increased thiol, SOD and CAT values as well as lymphocytes percentage and also treatment with its highest concentration (0.5 mg/mL) caused reduction of neutrophils and eosinophils percentages (*p *< 0.05 to *p *< 0.001), (Figures 1-7). There were still significant differences in thiol, SOD, CAT, and total WBC values between control group and sensitized animals treated with all concentrations of rosmarinic acid (*p *< 0.05 to *p *< 0.001), (Figures 3-6). MDA value due to treatment of two lower concentrations (*p *< 0.001 and *p *< 0.01 for 0.125 and 0.25 mg/mL respectively) as well as NO_3_ value (*p *< 0.001), neutrophils (*p *<0.01) and lymphocytes (*p *< 0.01) percentages due to treatment with low concentration of rosmarinic acid (0.125 mg/mL) and NO_2_ value (*p *< 0.001) due to treatment of its high concentration (0.5 mg/mL) were also significantly different with those of control group (Figures 1 and 7). 

Statistical significance for the differences between the data of S, S + D, S + O and S + R versus C: *: *p*<0.05, ***:* p*<0.001.

Statistical significance for the differences between the data of S + D, S + O and S + R versus S: ++: *p *< 0.01, +++: *p *< 0.001.

*Statistical significance for the differences between the data of S + O and S + R versus S + D: #: *p *< 0.05, ##: *p *< 0.01. The statistical comparisons were made using (ANOVA) with Tukey-Kramer’s post-test.*

Statistical significance for the differences between the data of S, S + D, S + O and S + R versus C: *: *p *<0.05, **: *p *<0.01, ***: *p *<0.001.

Statistical significance for the differences between the data of S + D, S + O and S + R versus S: +: *p *< 0.05, ++: *p *<0.01, +++: *p *<0.001.

*Statistical significance for the differences between the data of S + D versus S + O and S + R: #: *p *<0.05, ##: *p *<0.01, ###: *p *<0.001. The statistical comparisons were made using (ANOVA) with Tukey-Kramer’s post-test.*

*Differences between the effects of three concentrations of the extract and rosmarinic acid*

The effects of two higher concentrations of the extract (1.5 and 3.0 mg/mL) on serum concentrations of all oxidant and antioxidant biomarkers except of SOD in treated group with medium concentration of the extract were significantly higher than the effect of treatment with its low concentration (0.75 mg/mL), (*p *< 0.01 to *p *< 0.001). The effect of high concentration of the extract (3.0 mg/mL) on serum concentrations of MDA, thiol, SOD, and CAT were also significantly greater than its medium concentration (*p* < 0.001 for all cases), (Table 1).

**Table 1:** Serum concentrations of NO_2_, NO_3_, MDA, thiol, SOD and CAT in control rats (C), sensitized animals (S), S treated with dexamethasone (S + D), three concentrations of *O. basilicum* (S + O) and three concentrations of rosmarinic acid (S + R), (n = 6 for R treated groups and n = 8 for other groups)

**Mediators**	**NO2 **(**μM**)	**NO3 **(**μM**)	**MDA **(**nM**)	**Thiol **(**μM**)	**SOD **(**U/mL**)	**CAT **(**U/mL**)
**S + O0.75**	3.55±0.06	18.25±0.81	0.95±0.017	0.038±0.003	0.009±0.0001	0.013±0.0003
**S + O1.50**	3.19±0.05++	12.42±0.62+++	0.57±0.003+++	0.063±0.001+++	0.01±0.0001	0.031±0.0001+++
**S + O3.00**	3.08±0.1++	11.58±0.47+++	0.44±0.007+++###	0.088±0.003+++###	0.022±0.002+++###	0.08±0.0001+++###
**S +R0.125**	2.92±0.08**	16.62±0.35	0.84±0.018***	0.026±0.0008*	0.008±0.0001	0.012±0.0006
**S +R0.250**	2.61±0.14**	10.46±0.63+++	0.43±0.002***+++	0.09±0.002***+++	0.014±0.001	0.06±0.0004***+++
**S +R0.500**	1.15±0.17***+++###	7.03±0.39***+++###	0.32±0.03***+++##	0.13±0.002***+++###	0.031±0.004*+++###	0.09±0.0001***+++###

Values are presented as mean ± SEM. Three concentrations of *O. basilicum* were 0.75, 1.50 and 3.00 mg/mL, three concentrations of rosmarinic acid were 0.125, 0.250 and 0.500 mg/mL and that of dexamethasone was 1.25 μg/ mL.

Statistical significance for the differences between the data of S + R versus S + O:

Statistical significance for the differences between the data of S + O1 5 and S + O3.0 versus S + O0.75, and also S + R0.25 and S + R0.5 versus S + R0.125:

Statistical significance for the differences between the data of S + O3.0 versus S + O1.5 and S + R0.5 versus S + R0.25:

* : *p *< 0.05,

** : *p *< 0.01,

*** : *p *< 0.001.

++ : *p *< 0.01,

+++ : *p *< 0.001.

## : *p *< 0.01,

### : *p *< 0.001. The statistical comparisons were made using (ANOVA) with Tukey-Kramer’s post-test.

There were greater improvement in all differential WBC number due to high extract concentration (3.0 mg/mL) treatment and in neutrophils, monocytes, and lymphocytes percentages due to medium extract concentration (1.5 mg/mL) treatment compared to treated group with its low concentration (*p *< 0.01 to *p *< 0.001), (Table 2).

**Table 2:** Values of blood total and differential WBC count in control rats (C), sensitized animals (S), S treated with dexamethasone (S + D), three concentrations of *O. basilicum* (S + O) and three concentrations of rosmarinic acid (S + R), (n = 6 for R treated groups and n = 8 for other groups)

**Groups**	**Total WBC**	**Eosinophil**	**Monocyte**	**Neutrophil**	**Lymphocyte**
**S + O0.75**	7881.25±638.07	3.12±0.35	4.25±0.36	30±1.08	62.25±1.80
**S + O1.50**	7600±355.32	2.62±0.53	2.25±0.36++	24.25±1.81+	70.87±1.20++
**S + O3.00**	7412.5±646.82	2±0.42+	2±0.26+++	19.75±0.75+++	76.25±1.66+++
**S +R0.125**	4941.6±972.23*	2.83±0.60	3.83±0.30	27.5±1.38	65±1.48
**S + R0.250**	4658.33±791.98*	2.5±0.42	2±0.25++	23.33±1.45	72.16±1.42++
**S + R0.500**	4791.6.3±661.49	0.66±0.33*+##	2±0.36++	20±1.23++	77.33±1.02+++#

Values are presented as mean ± SEM. The data of WBC is their count in 1 mL blood and those of each type of WBC is the percentage of total WBC. Three concentrations of *O. basilicum* (O) were 0.75, 1.50 and 3.00 mg/mL, three concentrations of rosmarinic acid were 0.125, 0.250 and 0.500 mg/mL and that of dexamethasone was 1.25 μg/mL.

Statistical significance for the differences between the data of S + R versus S + O:

Statistical significance for the differences between the data of S + O1 5 and S + O3.0 versus S + O0.75, and also S + R0.25 and S + R0.5 versus S + R0.125:

Statistical significance for the differences between the data of S + O3.0 versus S + O1.5 and S + R0.5 versus S + R0.25:

* : *p *< 0.05.

+ : *p *< 0.05,

++ : *p *< 0.01,

+++ : *p *< 0.001.

# : *p *< 0.05,

## : *p *< 0.01. The statistical comparisons were made using (ANOVA) with Tukey-Kramer’s post-test.

The effects of high concentration of rosmarinic acid (0.5 mg/mL) on serum concentrations of all oxidant and antioxidant biomarkers and the percentage of all differential WBC, the effects of its medium concentration (0.25 mg/mL) on serum concentrations of NO_3_, MDA, thiol and CAT as well as percentages of lymphocytes and monocytes were significantly greater than those of its low concentration (0.125 mg/mL), (*p *< 0.05 to *p *< 0.001), (Tables 1 and 2). The effects of high concentration of rosmarinic acid on serum concentrations of all oxidant and antioxidant biomarkers and the percentages of eosinophils and lymphocytes were significantly higher than its medium concentration (0. 25 mg/mL) (*p *< 0.05 to *p *< 0.001), (Tables 1 and 2).

*Differences between the effects of the extract, rosmarinic acid and dexamethasone*

The effects of all concentrations of extract on serum concentrations of NO_2_, MDA, and thiol, the effects of its two higher concentrations (1.5 and 3.0 mg/mL) on serum concentrations of CAT, the effects of its high concentration (3.0 mg/mL) on serum concentrations of NO_3_, SOD, and percentage of eosinophil as well as the effects of low and medium concentrations of the extract on total WBC were significantly lower than those of rosmarinic acid (*p *< 0.05 to *p *< 0.001) (Tables 1 and 2). 

The effects of treated groups with all concentrations of extract on serum concentrations of MDA and thiol, the effects of treated groups with its two lower concentrations on SOD and CAT and also the effects of treated groups with its low concentration on NO_2_ were significantly lower than dexamethasone treatment (*p *< 0.05 to *p* < 0.001), (Figures 1-5). However, serum concentration of NO_3_ was improved significantly higher in treated groups with two higher concentrations of the extract compared to dexamethasone treatment (*p *< 0.05 and *p *< 0.001 for 1.5 and 3.0 mg/mL of the extract concentrations respectively), (Figure 1). 

The effects of all concentrations of rosmarinic acid on serum concentration of thiol, its two lower concentrations on MDA and CAT and its low concentration on SOD were lower than dexamethasone treatment (*p *<0.001 for all cases), (Figures 2-5). However, the effects of all concentrations of rosmarinic acid on total WBC count, its two higher concentrations on NO_2_ and NO_3_ as well as its highest concentration on SOD were higher than the effect of dexamethasone treatment (*p *< 0.05 to *p *< 0.001), (Figures 1, 4 and 6). 

## Discussion

The effects of *O. basilicum *and its constituent, rosmarinic acid, on total and differential blood WBC count, serum levels of NO_2_, NO_3_, MDA, thiol, SOD and CAT in sensitized rats were examined in the present study. 

Serum concentrations of oxidant biomarkers, total WBC count and percentages of eosinophils and neutrophils in blood of untreated sensitized group were significantly higher, but antioxidant biomarkers and percentage of lymphocyte were significantly less than those of control group. These results confirmed the sensitization of animals. In fact previous studies showed that asthma is associated with a strong oxidant stress that is a result of both increased oxidant systems and decreased antioxidant capacity (27). Oxidants are produced at higher amounts in patients with asthma compared with healthy subjects (4). Higher levels of NO, the main nitrogen species produced in the lung (28) and malondialdehyde, a marker of lipid peroxidation (29) are correlated with the severity of the asthma. In addition, asthma is characterized by the loss of antioxidant activities (27). Studies showed that under oxidative stress, activities of thiol (30) catalase (31) and SOD (32) are decreased. The increased total WBC and eosinophil count in sensitized animals (33) and asthmatic patients (7) were also documented. Increased eosinophil can release a variety of mediators including eosinophil cationic protein, major basic protein (34) eosinophil derived neurotoxin, eosinophil peroxidase, superoxide ion, and lipoxin A. Increased lymphocytes can also recognize antigen through specific receptors and thereby initiate an inflammatory response by releasing cytokines (35). Therefore, the results confirmed the sensitization of rats in the present study.

The results showed that treatment of sensitized animals with the extract of *O. basilicum* and rosmarinic acid leads to decrease of all measured parameters except antioxidant biomarkers (thiol, SOD and CAT) and lymphocytes percentage which were increased in treatment groups. These results suggest that extract and rosmarinic acid have anti-oxidant and anti-inflammatory properties. Therefore, the plant extract may have preventive effects on inflammatory diseases via reduction of involved cells in the respiratory tract inflammation. Anti-oxidant and anti-inflammatory effects of *O. basilicum* and rosmarinic acid have been shown previously which support the results of the current study. Antioxidant activity of *O. basilicum *was shown by isolation of potential antioxidant compounds of this plant (36). Another study indicated that the extract reduces the reproductive alterations induced by an organophosphorus insecticide (diazinon) with serious oxidative effects, in albino rats which may be due to the potent antioxidant effects of the extract components (37). Antioxidant capacity of basil extract can also decrease oxidative effects of electromagnetic field on neural cells of rats (38). In addition, anti-inflammatory activity of the alcoholic extract of *O. basilicum *in human peripheral blood mononuclear cells (PBMC) and its inhibitory effect on proinflammatory cytokines and mediators was reported (39). The ethanolic extract of this plant also decreased the inflammatory reaction (paw edema) induced by carrageenan in male Wistar rats which could be related to its antioxidant effects (40). Lee KG and colleagues showed that the major antioxidant compound of basil was rosmarinic acid. The antioxidant activity of rosmarinic acid in the liposome system was also shown (16). It was also showed that the anticarcinogenic effect of *P. frutescens* extract was partially due to anti-inflammatory and antioxidant property of rosmarinic acid (41).

The reduction of lymphocyte percentage in sensitized rats is in fact due to increased total WBC count. In a previous study, increased absolute number of the lymphocyte in sensitized animals using similar method of sensitization was reported (42). In addition, increased lymphocyte percentage in treated groups is also due to reduction of total WBC count. 

The results of this study also showed concentration dependent preventive effect of the extract and rosmarinic acid on sensitized animals. The effects of low concentrations of extract and rosmarinic acid on all measured parameters except total WBC count were less than the effects of its high concentrations. The effects of medium concentration of the extract on all measured parameters except SOD, total WBC, and eosinophils percentage were also significantly higher than the effect of treatment with its low extract concentration. The effects of medium concentration of rosmarinic acid on serum concentrations of NO3, MDA, thiol, CAT, and percentage of lymphocytes and monocytes were significantly greater than those of its low concentration. The effects of high concentration of the extract on serum concentrations of MDA and all antioxidant biomarkers were significantly higher than its medium concentration. The effects of high concentration of rosmarinic acid on serum concentrations of all oxidant and antioxidant biomarkers and the percentages of eosinophils and lymphocytes were significantly higher than the effects of its medium concentration. The concentration dependency effect of the extract and rosmarinic acid also could be another evidence for their antioxidant and anti-inflammatory effects.

Furthermore, the findings of the present study also suggest that the antioxidant and anti-inflammatory effects of the extract is perhaps partially due to its constituent, rosmarinic acid. The effects of all concentrations of the extract on serum concentrations of NO_2_, MDA and thiol, the effects of its two higher concentrations on serum concentrations of CAT, and the effect of its high concentration on serum concentrations of NO_3_, SOD and percentage of eosinophils and the effects of low and medium concentrations of the extract on total WBC count were significantly lower than those of rosmarinic acid. However, it should be noted that the concentrations of rosmarinic acid used in the present study were one sixth (1/6) or 16% of those of the extract (0.125, 0.25 and 0.5 mg/mL for rosmarinic acid and 0.75, 1.5 and 3.0 mg/mL for the extract). The HPLC results of the previous study indicated that rosmarinic acid level was about 10 µg/mg plant dry weight which means 1% of the dry plant (43). Therefore, the actual concentration of rosmarinic acid in the plant is less than its concentrations used in this study. These results suggest that rosmarinic acid only partially responsible for antioxidant and anti-inflammatory effects of *O. basilicum* extract which also supported by a previous study (41).

Comparable effect of extract of *O. basilicum* and rosmarinic acid, with that of dexamethasone is another important evidence indicating their antioxidant and anti-inflammatory effects. In fact, the inhibitory effect of dexamethasone on respiratory tract inflammation and its effect on lymphocytes in asthmatic mice have been shown, which support the results of the present study (44). Serum concentration of NO_3_ was improved significantly higher in treated groups with two higher concentrations of the extract compared to dexamethasone treatment. The effects of all concentrations of rosmarinic acid on total WBC count, its two higher concentrations on NO_2_ and NO_3_ and its high concentration on SOD were higher than the effects of dexamethasone treatment. These results showed comparable or even more potent antioxidant and anti-inflammatory effects of the extract of *O. basilicum *and its constituent rosmarinic acid compared to the dexamethasone effects at used concentrations. 

The present study together with other studies indicate anti-inflammatory and antioxidant effects of *O. basilicum* and rosmarinic acid in sensitized rats which suggest a therapeutic effect on inflammatory diseases such as asthma by reducing oxidant stress and inflammation. However further studies needed to evaluate the effects of this plant and its constituents on asthmatic patients. In addition, the antioxidant and anti-inflammatory effects of other constituents of *O. basilicum* should be also evaluated in further studies. 

## Conclusion

The results of this study showed a preventive effect of the extract of *O. basilicum *and its constituent, rosmarinic acid on serum levels of NO_2_, NO_3_ and MDA , total and differential WBC in the blood of sensitized rats which was comparable or even more potent than dexamethasone at used concentrations. The results also suggest that the effect of the plant is perhaps partially due to its constituent, rosmarinic acid. These results may suggest the potential therapeutic effect of this plant and its constituent, rosmarinic acid on inflammatory diseases such as asthma.
